# Synthesis and photoelectrochemical response of CdS quantum dot-sensitized TiO_2_ nanorod array photoelectrodes

**DOI:** 10.1186/1556-276X-8-222

**Published:** 2013-05-10

**Authors:** Yunxia Hu, Baoyuan Wang, Jieqiong Zhang, Tian Wang, Rong Liu, Jun Zhang, Xina Wang, Hao Wang

**Affiliations:** 1 Faculty of Physics and Electronic Technology, Hubei University, Wuhan, 430062, People’s Republic of China

**Keywords:** Quantum dots, CdS, Nanocable arrays, SILAR

## Abstract

A continuous and compact CdS quantum dot-sensitive layer was synthesized on TiO_2_ nanorods by successive ionic layer adsorption and reaction (SILAR) and subsequent thermal annealing. The thickness of the CdS quantum dot layer was tuned by SILAR cycles, which was found to be closely related to light absorption and carrier transformation. The CdS quantum dot-sensitized TiO_2_ nanorod array photoelectrodes were characterized by scanning electron microscopy, X-ray diffraction, ultraviolet–visible absorption spectroscopy, and photoelectrochemical property measurement. The optimum sample was fabricated by SILAR in 70 cycles and then annealed at 400°C for 1 h in air atmosphere. A TiO_2_/CdS core-shell structure was formed with a diameter of 35 nm, which presented an improvement in light harvesting. Finally, a saturated photocurrent of 3.6 mA/cm^2^ was produced under the irradiation of AM1.5G simulated sunlight at 100 mW/cm^2^. In particular, the saturated current density maintained a fixed value of approximately 3 mA/cm^2^ without decadence as time passed under the light conditions, indicating the steady photoelectronic property of the photoanode.

## Background

The quantum dot-sensitized solar cell, which may be considered as the third generation of solar cells, has attracted great scientific and industrial interest in recent years [1-3]. Inorganic quantum dots (QDs), such as CdS [4-6], CdSe [7,8], and CdTe [9], have the following advantages as sensitizers: an effective bandgap controlled by the size of the QDs, large absorption of light in the visible region, and the possibility for multiple exciton generation. Among the various QD materials, CdS has been receiving much attention because of its high potential in photoabsorption in the visible region. Thus, CdS has been widely studied and applied to light-emitting diodes [10], biology applications [11], and solar cells [12,13]. CdS QDs are prepared using several methods, including thermal evaporation [14], spray pyrolysis [15], chemical bath deposition (CBD) [16], and successive ionic layer adsorption and reaction (SILAR) [17]. Among these methods, SILAR is the most commonly used given its simple technique and capacity to produce high-quality nanoparticles in large scale.

One-dimensional (1D) single-crystalline oxide array is very popular because of its higher specific surface area than that of its film, its ability to grow easily over a large area on the substrate, as well as its bandgap that can match well with CdS. Several studies on 1D single-crystalline oxide array have been reported [18,19]. Yao et al. [18] reported on CdS QD-sensitized ZnO nanorod arrays (NRAs) that displayed a power conversion efficiency of 1.07%. CdS QD-sensitized TiO_2_ NRA solar cells have been prepared through the CBD method with a photocurrent intensity of 5.13 mA/cm^2^ at 0-V potential and an open-circuit potential of −0.68 V [19]. We have synthesized various sizes of CdS QDs and dye-co-sensitized TiO_2_ NRA solar cells by SILAR, yielding a power conversion efficiency of 2.81% [20]. In the present study, the photoelectrochemical properties and stability of the TiO_2_/CdS core-shell NRA photoelectrode were studied. In our experiment, TiO_2_ nanorods were prepared through the hydrothermal method without a seed layer, and the CdS QDs were synthesized by SILAR. The optimum CdS QD-sensitized TiO_2_ NRA photoelectrode that formed the TiO_2_/CdS core-shell structure with a shell thickness of 35 nm was fabricated by SILAR in 70 cycles and then annealed at 400°C for 1 h in air atmosphere. This photoelectrode presented an improvement in light harvesting, ultimately producing a saturated photocurrent of 3.6 mA/cm^2^ under the irradiation of AM1.5G simulated sunlight at 100 mW/cm^2^. In particular, the saturated current density maintains a fixed value of approximately 3 mA/cm^2^ without decadence as time passed under the light conditions, indicating the steady photoelectronic property of the photoanode.

## Methods

TiO_2_ NRAs were prepared through the hydrothermal method. Approximately 8 mL of deionized water was mixed with 8 mL of concentrated hydrochloric acid (36.5% to 38% by weight) to reach a total volume of 16 mL. The mixture was stirred in air for 5 min. Then, 0.2 mL of titanium butoxide was added into the solution, which was stirred for another 5 min. A fluorine-doped tin oxide (FTO) substrate (approximately 2 cm × 2 cm) was placed in a 20-mL autoclave. The hydrothermal method was used to grow the TiO_2_ NRAs at 150°C for 10 h. Samples were annealed at 500°C for 2 h in air. CdS QDs were deposited on the TiO_2_ nanorods through SILAR. The FTO substrate grown with TiO_2_ NRAs was immersed in a 0.3 mol/L Cd(CH_3_COO)_2_ aqueous solution for 2 min, rinsed with deionized water, then immersed for another 2 min in a 0.3 mol/L Na_2_S aqueous solution, and rinsed with deionized water. The above series of steps were carried out to prepare the CdS QDs, and these steps were repeated several times until a thin layer of quantum dots was formed. The samples were then annealed at 400°C for 1 h in air atmosphere.

The morphology of the sample was studied by scanning electron microscopy (FE-SEM; JEOL JSM-6700F, Akishima-shi, Japan). The structure and crystallinity of the samples were investigated by X-ray diffraction (XRD; D8, Bruker AXS, Inc., Madison, WI, USA). The optical properties of the samples were characterized by ultraviolet–visible (UV–vis)-IR absorption (UV360 spectrometer, Shimadzu, Corporation, Kyoto, Japan). The microstructure of a single nanorod was observed by transmission electron microscopy (TEM; FEI TECNAI G20, Hillsboro, OR, USA). Photoelectrochemical measurements were performed in a sulfide/polysulfide (S^2−^/Sn^2−^) electrolyte containing 0.5 M S and 0.3 M Na_2_S dissolved in deionized water, in which the TiO_2_/CdS arrays on FTO, Pt foil, and SCE were used as the working, counter, and reference electrodes, respectively. The illumination source used was AM1.5G light at 100 mW/cm^2^.

## Results and discussion

**Figure 1:**
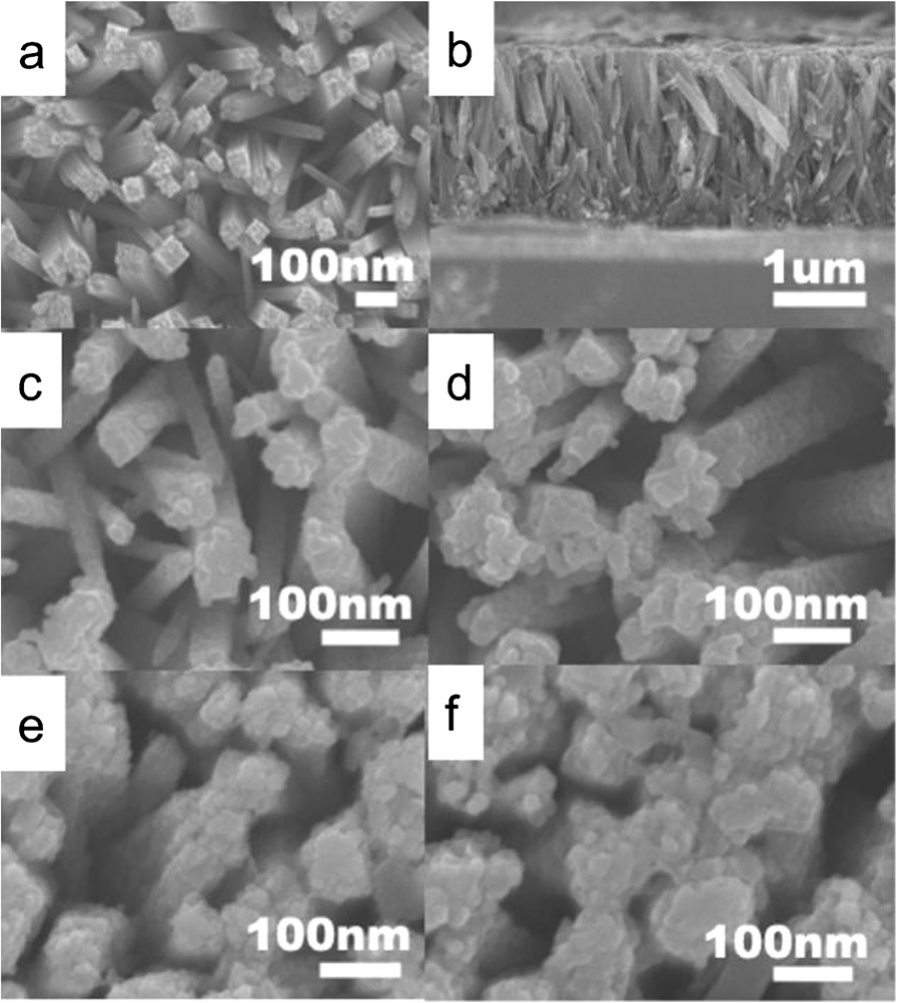
**SEM images of TiO**_**2 **_**nanorod arrays and TiO**_**2**_**/CdS core-shell structure with different cycles.** (**a**) Top view of bare TiO_2_ nanorod arrays. (**b**) Cross-sectional view of bare well-aligned TiO_2_ nanorod arrays. Top view of the TiO_2_/CdS core-shell structure with (**c**) 10, (**d**) 30, (**e**) 70, and (**f**) 80 SILAR cycles.

Figure 1 shows the SEM images of the TiO_2_ NRAs and the TiO_2_/CdS core-shell structure. The TiO_2_ NRAs are vertically aligned on the FTO, with an average diameter of 80 to 100 nm, as shown in Figure 1a. The TiO_2_ nanorods are dense and compactly arranged in the same direction. The top facets of the nanorods appear rough, and the side facets are smooth. In addition, the nanorods show a uniform length. The TiO_2_ NRAs are grown perpendicularly to the FTO substrate, with lengths of about 3 μm, which is helpful for QD sensitization, as shown in Figure 1b. CdS QDs are deposited on the TiO_2_ NRAs (denoted as FTO/TiO_2_/CdS) by SILAR. After the deposition of CdS QDs, the entire surface of the TiO_2_ NRAs was uniformly covered with dense CdS QDs. Moreover, the cycle times of CdS QDs increased (Figure 1c,d,e,f), the surface of TiO_2_ NRAs gradually became rough, and the diameter of TiO_2_/CdS was thicker. The diameters of the TiO_2_/CdS core-shell structure with 10, 30, and 70 cycles were approximately 90 to 110 nm, 125 to 150 nm, and 150 to 175 nm, respectively. The gap between the TiO_2_ nanorods became smaller.

**Figure 2:**
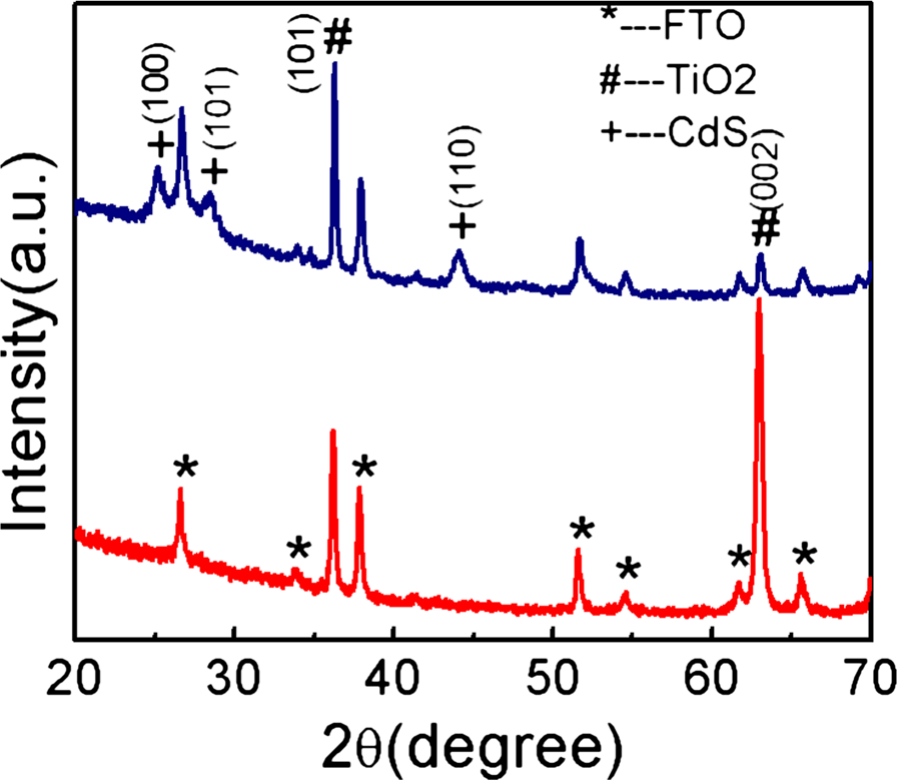
**XRD patterns of TiO**_**2 **_**nanorods (blue curve) and TiO**_**2**_**/CdS core-shell structure on FTO (red curve).**

Figure 2 shows the XRD patterns of the TiO_2_ NRAs (blue curve) and the TiO_2_/CdS core-shell structure (red curve). The XRD pattern showed that the TiO_2_ samples have a tetragonal rutile structure and the FTO substrates have a rutile structure (JCPDS no. 41-1445). Three peaks appeared at 36.2°, 62.9°, and 70.0°, which are respectively indexed to the (101), (002), and (112) planes of the TiO_2_ (JCPDS no. 89-4920). The enhanced (002) peak located at 62.9° indicates that the nanorods are well crystallized and grew along the (001) direction. After the deposition of CdS with a hexagonal structure (JCPDS no.06-0314), three diffraction peaks were related to CdS and located at 25.1°, 28.4°, 43.9°, corresponding to (100), (101), and (110), respectively. The XRD peaks of CdS are fairly broad, which indicates that the size of CdS nanoparticles is very small.

**Figure 3:**
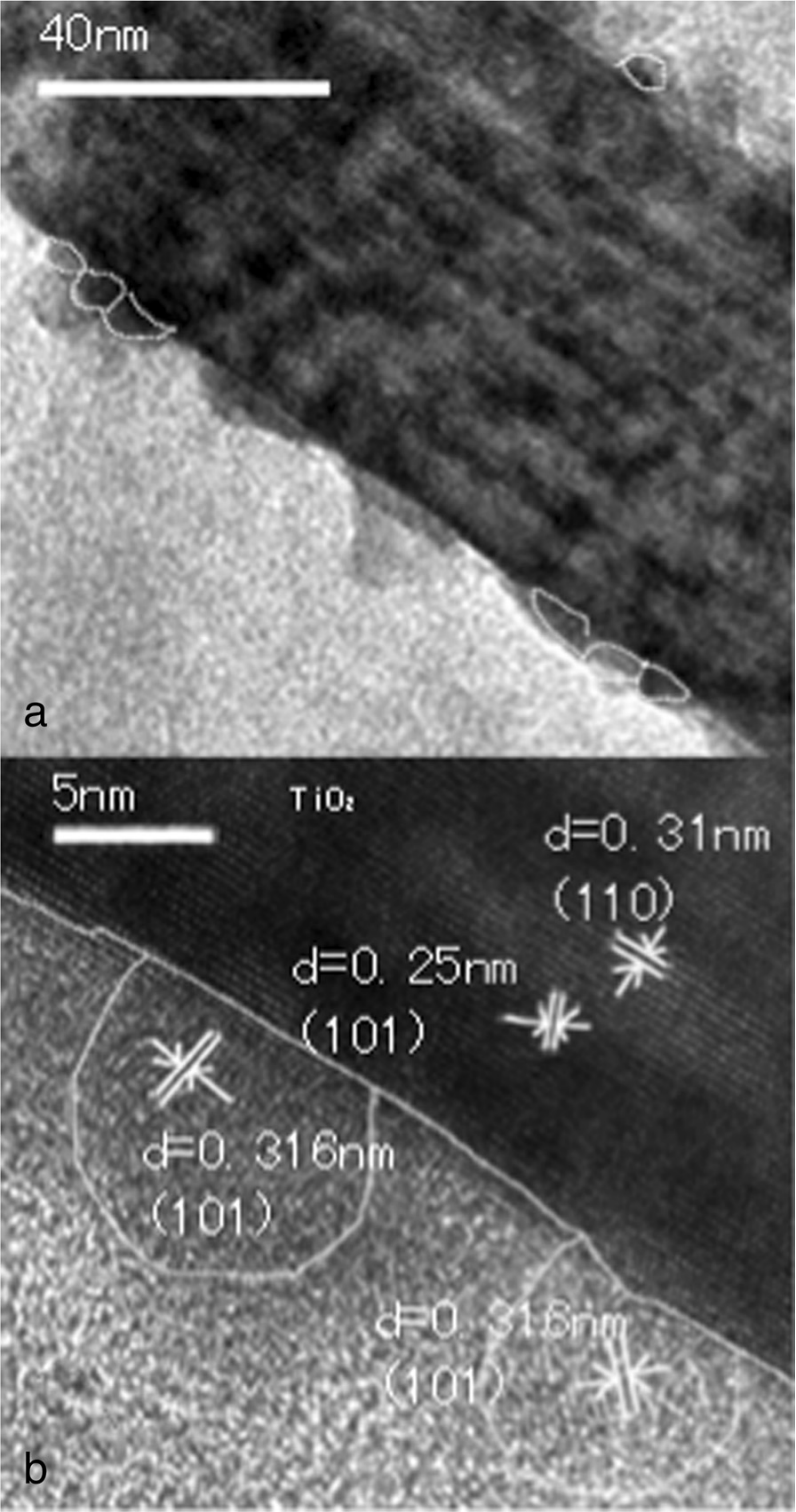
**TEM images of a single TiO**_**2**_**/CdS core-shell structure.** At (**a**) low magnification and (**b**) high resolution showing the TiO2/CdS interface.

Figure 3 shows the TEM structure of the TiO_2_/CdS core-shell structure and the high-resolution TEM image. The typical TEM image of the TiO_2_/CdS core-shell structure is shown in Figure 3a. The CdS nanoparticles with an average size of 3 to 7 nm were found to be attached to the surface of the TiO_2_ nanorod compactly, which is in the range of the exciton Bohr radius of CdS. Thus, the sizes of the CdS on the TiO_2_ NRAs in our work are still within the QD scale. Based on the HRTEM images captured from different regions of the TiO_2_/CdS core-shell structure, clear interfaces were formed between the CdS QDs and the TiO_2_ core. The observed lattice spacing of 0.31 and 0.25 nm in the ‘core’ region correspond to the (110) and (101) phases of tetragonal rutile TiO_2_ (JCPDS no. 89-4920). The lattice fringe spacing of 0.31 nm for each nanoparticle in the ‘shell’ matches well to the interplanar space of the (101) phase of CdS (JCPDS no. 06-0314), indicating that the shell is composed of a single-crystalline CdS QD with different orientation.

**Figure 4:**
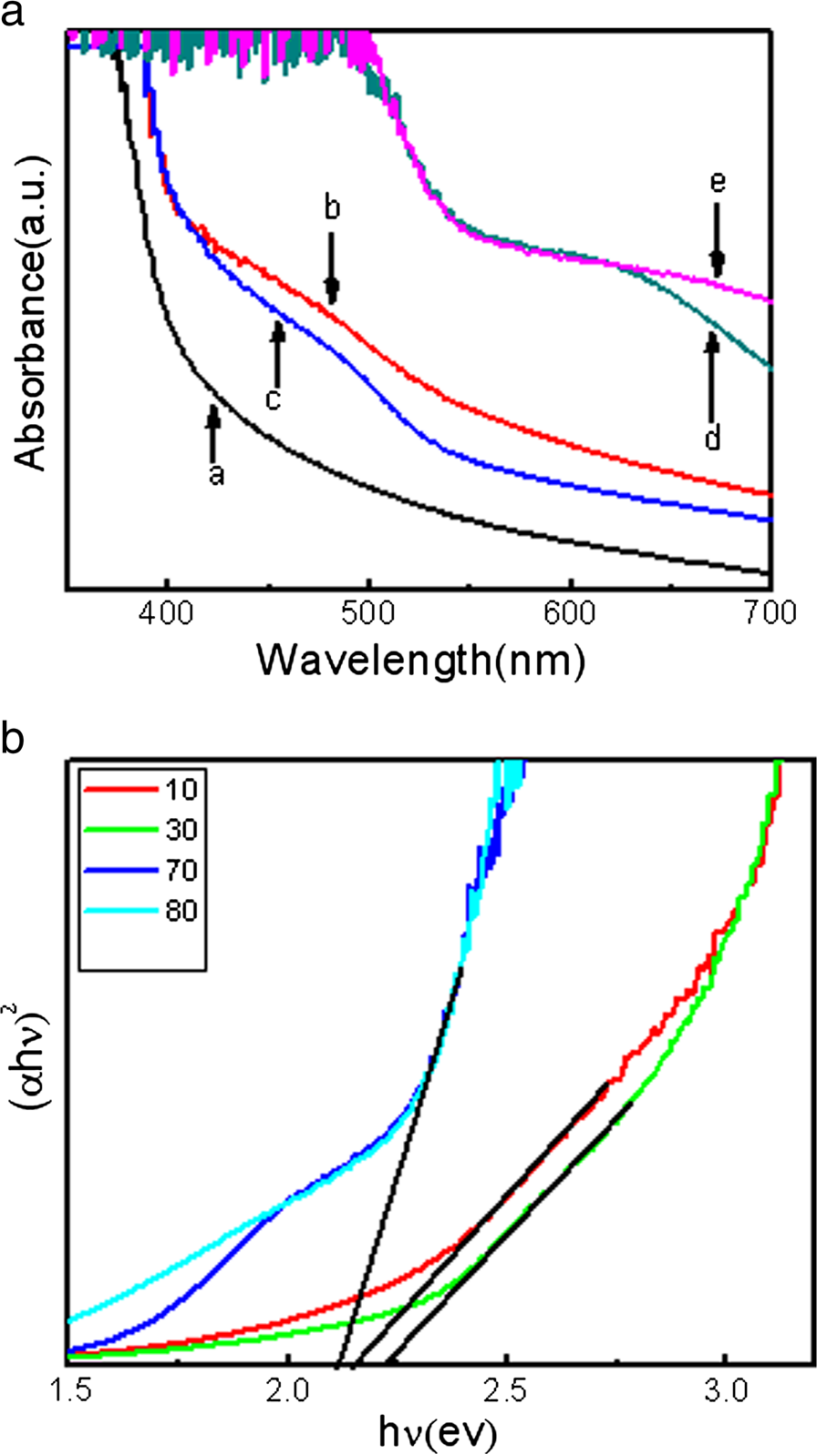
**UV–vis absorption spectra and Tauc analysis of (*hνα*)**^**2 **^**versus *hν *plots.** (**a**) UV–vis absorption spectra of TiO_2_ nanorod arrays and TiO_2_/CdS core-shell structure with different cycles: (a) TiO_2_ nanorods and TiO_2_/CdS core-shell structure with (b) 10, (c) 30, (d) 70, and (e) 80 SILAR cycles. (**b**) Tauc analysis of (*hνα*)^2^ versus *hν* plots derived from the absorption spectra.

Figure 4a shows the typical absorption spectra of the TiO_2_ nanorods and the TiO_2_/CdS core-shell structure electrodes. The absorption edge of the TiO_2_ appears at 380 nm. The absorption edge of the CdS QD-sensitized TiO_2_ NRAs red-shifted at 514 nm, which is close to the bandgap of CdS (approximately 2.41 eV). The absorption intensity was enhanced with the increase of the CdS QD quantity on TiO_2_, and the absorption edge gradually moved to a longer wavelength in the entire UV–vis region. The result indicates that the TiO_2_/CdS core-shell structure has better optical performance. The exact bandgap values can be obtained by employing a Tauc analysis of (*hνα*)^2^ versus *hν* plots derived from the absorption spectra. As shown in Figure 4b, the extrapolation of the linear part until its intersection with the *hν* axis provides the value of the bandgap, which is determined as 2.1 to 2.3 eV for CdS particles with different cycles. Compared with the values of bulk CdS (2.4 eV), the sizes of the CdS in the present work are still within the QD scale.

**Figure 5:**
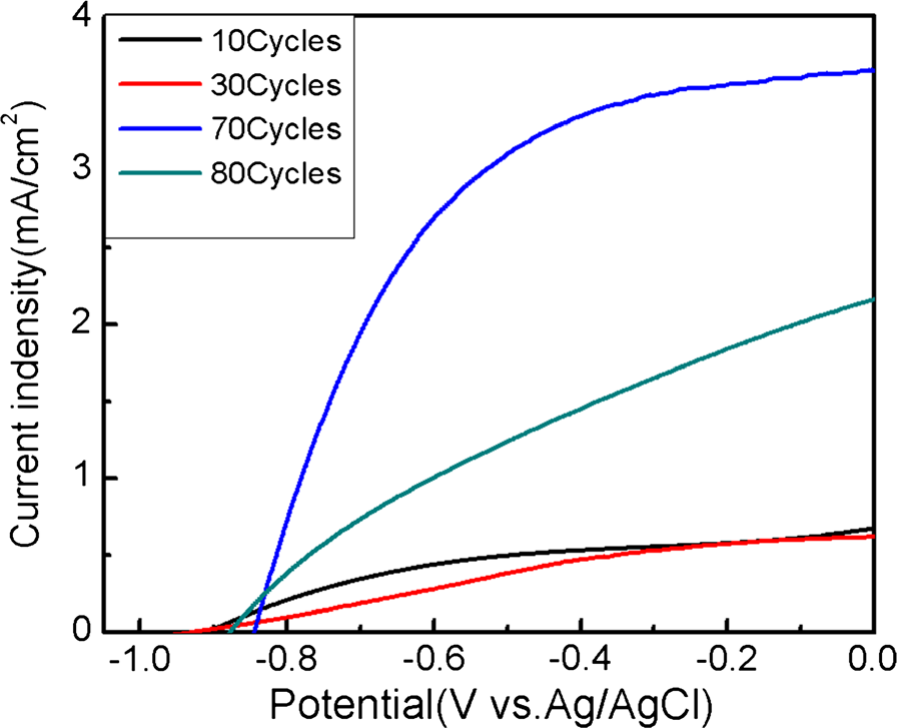
**Different current densities versus potential curves.** TiO_2_/CdS photoelectrodes with different cycles measured under illumination of AM1.5G light at 100 mW/cm^2^: 10 (black curve), 30 (red curve), 70 (blue curve), and 80 (green curve) cycles.

Figure 5 shows the photocurrent density versus potential characteristics of the TiO_2_/CdS core-shell structure in different cycles. With the increase in the number of cycles, the photocurrent density initially becomes larger before decreasing at 80 cycles. This trend could be explained by the excess CdS QDs that filled the gaps within the nanocrystalline TiO_2_ nanorods, which led to the decrease in the contact area between the CdS QDs and the electrolyte. Simultaneously, the excess CdS QDs resulted in the increase of electron recombination among the CdS QDs. From the saturated blue curve in Figure 5, the optimal number of cycles was 70, which displays the ideal current density of 3.6 mA/cm^2^.

**Figure 6:**
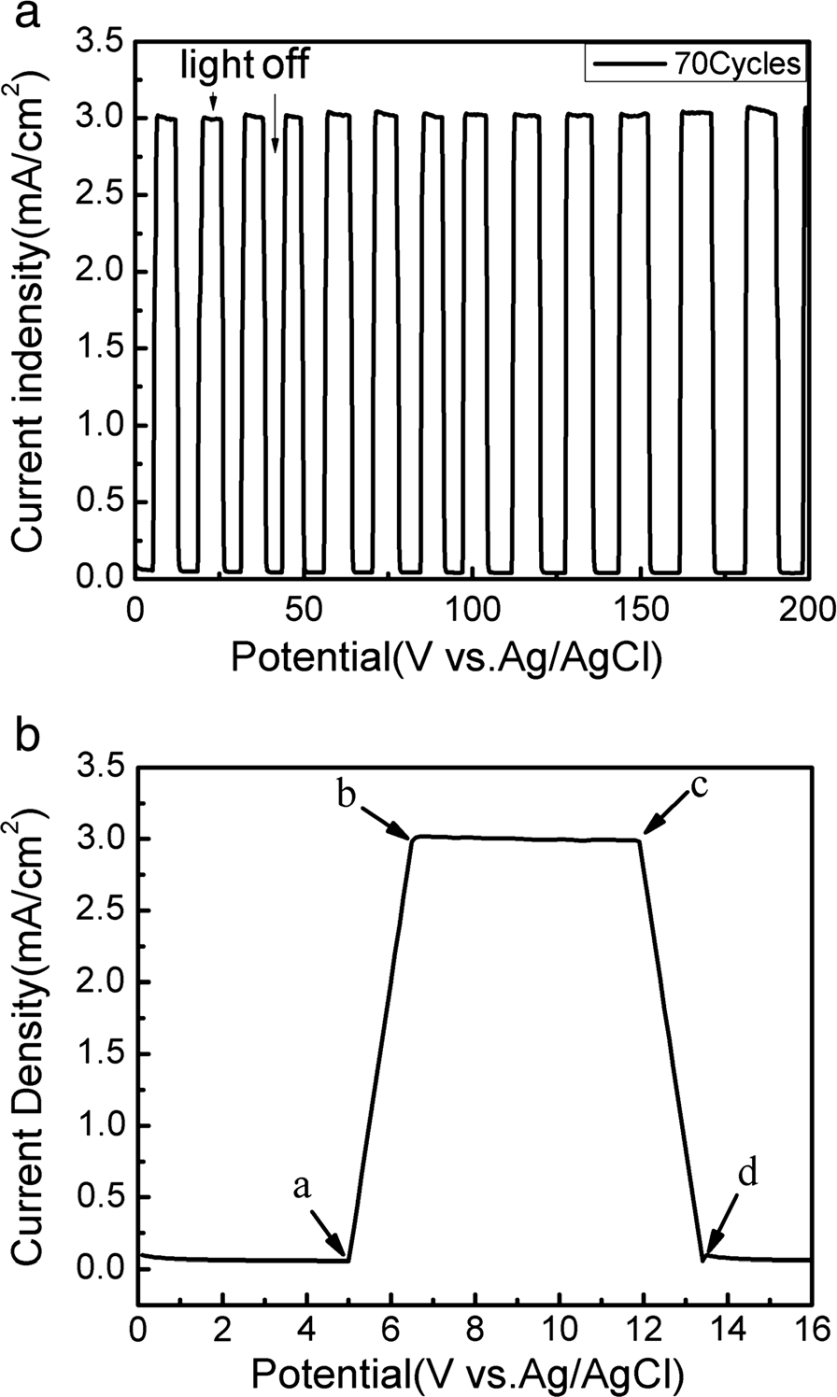
**Current density-time curve and the enlarged portion of the photocurrent response.** (**a**) Current density-time curve of the TiO_2_/CdS core-shell structure with 70 SILAR cycles at sunlight illumination (AM1.5G, 100 mW/cm^2^). (**b**) The enlarged portion of the photocurrent response.

As an important characteristic, solar cell stability is an essential factor in QD solar cells for industrialization. Therefore, the photocurrent response curve of the device was plotted to characterize the stability of the device. Figure 6 shows the corresponding photocurrent response curve of the device with 70 cycles of CdS QDs. As shown in Figure 6a, the device is very stable, and its largest photocurrent density changes slightly when the device is under the irradiation of AM1.5G simulated sunlight at 100 mW/cm^2^. This result indicates that the device has steady photoelectrochemical performance in the polysulfide electrolyte, which is beneficial for optoelectronic device applications. Figure 6b shows a magnified area of the photocurrent response, including the fast-rise region (from a to b), saturation region (from b to c), and recovery region (from c to d). In the fast-rise region, the current density increased from 0.5 to 3.0 mA/cm^2^ within 1.5 s under the light and then remained constant. Upon light removal, the current density approached the recovery region, and the photocurrent decreased sharply to 0.5 mA/cm^2^. As a consequence, the TiO_2_/CdS core-shell structure devices showed excellent stability and fast response. Thus, this structure can be a promising application in solar cells as a photoelectrode.

## Conclusions

A simple SILAR method was used to prepare a CdS shell on TiO_2_ NRAs. The optimum sample was fabricated by SILAR in 70 cycles and then annealed at 400°C for 1 h in air atmosphere, providing an improvement of light harvesting and ultimately yielding a saturated photocurrent of 3.6 mA/cm^2^ under the irradiation of AM1.5G simulated sunlight. In particular, the saturated current density maintains a fixed value of about 3 mA/cm^2^ without decadence as time passed under the light conditions, indicating the steady photoelectronic property of the photoanode.

## Competing interests

The authors declare that they have no competing interests.

## Authors’ contributions

YH carried out the material and device preparation and drafted the manuscript. BW carried out the device characterization. JZ participated in the drafting of the manuscript. TW participated in the device preparation. RL carried out the optical absorption characterization. JZ participated in the revision of the manuscript. XW carried out the TEM observation. HW conceived of the study and participated in its design and coordination. All authors read and approved the final manuscript.
